# The potential role of local pharmacies to assess asthma control: an Italian cross-sectional study

**DOI:** 10.1186/s12889-020-10080-1

**Published:** 2021-01-05

**Authors:** M. Caminati, L. Cegolon, M. Bacchini, N. Segala, A. Dama, C. Bovo, B. Olivieri, F. Furci, G. Senna

**Affiliations:** 1 Department of Medicine, University of Verona, Verona, Italy; 2 Local Health Unit N.2 “Marca Trevigiana”, Public Health Department, Treviso, Italy; 3 Pharmacists’ Association of Verona, Verona, Italy; 4 Asthma Centre & Allergy Unit, Verona University Hospital, Verona, Italy; 5 Medical Directorate, Verona University Hospital, Verona, Italy; 6 Residency Programme in Allergy & Clinical Immunology, Verona University Hospital, Verona, Italy; 7 Department of Clinical & Experimental Medicine, University Hospital G. Martino, Messina, Italy

**Keywords:** Community pharmacies, Asthma, Control, Asthma control test, Treatment compliance

## Abstract

**Background:**

Asthma control and monitoring still represents a challenge worldwide. Although the international guidelines suggest the interplay between secondary and primary care services as an effective strategy to control the disease, community pharmacies’ are seldom involved in asthma control assessment.

The present cross-sectional study aimed at providing a picture of the relationship between asthma severity and control in community pharmacies within the health district of the city of Verona (Veneto Region, North-Eastern Italy).

**Methods:**

A call for participation was launched through the Pharmacists’ Association of Verona. Patients referring to the participating pharmacies with an anti-asthmatic drug medical prescription and an asthma exemption code were asked to complete the Asthma Control Test (ACT) and a brief questionnaire collecting information on their age, sex, smoking status, aerobic physical exercise and usual asthma therapy, which also defined asthma severity. A multinomial logistic regression model was fitted to investigate the risk of uncontrolled as well as poorly controlled vs. controlled asthma (base). Results were expressed as relative risk ratios (RRR) with 95% confidence interval (95%CI).

**Results:**

Fifty-seven community pharmacies accepted to participate and 584 asthmatic patients (54% females; mean-age: 51 ± 19 years) were consecutively recruited from 1st January to 30th June 2018 (6 months). Based upon ACT score 50.5% patients had a controlled asthma, 22.3% a poorly controlled and 27.2% uncontrolled. A variable proportion of patients with uncontrolled asthma were observed for every level of severity, although more frequently with mild persistent form of asthma. Most patients (92%) self-reported regular compliance with therapy. At multinomial regression analysis, patients under regular asthma treatment course (RRR = 0.33; 95%CI: 0.15; 0.77) were less likely to have an ACT< 16 compared to those not taking medications regularly.

**Conclusions:**

Overall, our findings highlighted an unsatisfactory asthma control in the general population, independently of the severity level of the disease. Community pharmacies could be a useful frontline interface between patients and the health care services, supporting an effective asthma management plan, from disease assessment and monitoring treatment compliance to referral of patients to specialist medical consultancies.

## Background

Asthma is one of the most common chronic disorders on a global scale, with a prevalence in the general population estimated to range from 1 to 18% [1–3].

The burden of asthma includes considerable financial impact in terms of direct (health care services, medications) and indirect (sickness absence from work, disability, other) costs [1, 2, 4].

The most striking contradiction in asthma is a general lack of its control [5–8], even for milder forms, despite the availability of very effective drugs which have been proven to be effective in most patients, if regularly taken [9].

Several countries have been adopting national plans to manage asthma, with the aim to improve its control and contain its impact, yet with unsatisfactory health outcomes [1, 10–12]. The involvement of primary care services in asthma management has been suggested as a successful strategy to improve the control of the disease, since they frequently represent the first point of contact for patients affected by chronic conditions [1, 13]. In particular, in several countries the involvement of community pharmacies has already proven to offer an efficient support for the management of chronic conditions such as diabetes, hypertension and, with more limited evidence, asthma [14]. Nonetheless, local pharmacies are still largely underused to promote health in the general population in high-income countries [15].

Usually patients have good relationships with their local pharmacists, they rely on them and are more comfortable in such health care setting than in a medical environment. Moreover, pharmacists are more easily accessible for patients, also because an appointment is not required. As a result, local pharmacies may be extremely relevant in promoting behavioural changes aimed at improving healthy lifestyles and treatment adherence to medications for various conditions, which considerably impacts on health care costs for national health services (NHS) [15]. Furthermore, pharmacists can contribute to disease control assessment and medical referral if need be.

### Aim of the study

In view of the above, the present cross-sectional study aimed at assessing the severity and control of asthma in community pharmacies within the health district of the city of Verona (Veneto region, North-Eastern Italy).

## Methods

### Ethical approval

The Ethic Committee of the Pharmacists’ Association of Verona Health District approved the study protocol. Written informed consent was obtained from all study participants.

#### Community pharmacies

By expanding a previous pilot study [16], a call for participation was launched through the local Pharmacists’ Association, which includes all the community pharmacies in the Verona health district. Before the study start, pharmacists attended a two-sessions seminar held by the Specialists of the Asthma Center – Allergy Unit at Verona University Hospital, which is recognised as a Referral Center for the diagnosis and treatment of asthma and allergic diseases. The seminar was organized as an interactive workshop on bronchial asthma, its management, the study design and study tools. A questionnaire was administered to each one of the participants in order to verify the acquisition of the essential information after the end of the second session. The study lasted 6 months, from the 1st of January 2018 to 30th of June 2018.

#### Patients

Within the study time frame, consenting patients referring to the participating pharmacies with an anti-asthmatic drug medical prescription and an asthma exemption code were consecutively recruited from 1st January to 30th June 2018 (6 months). The Italian NHS offers universal health care, with patients required to pay a subsidy for each health care service received (diagnostic, treatment or medicine). However, the Italian NHS assigns an exemption code to specific chronic health conditions, allowing affected patients to access health care services free of charge. The asthma exemption code (007–493) enables patients to access free health care and medications related to their asthma condition and is granted by the Italian NHS to individuals with a confirmed diagnosis of bronchial asthma, based on clinical history and lung function tests. The asthma exemption code therefore allows to accurately identify asthmatic patients.

Participating study subjects were asked to complete the Asthma Control Test (ACT, see below) and a brief questionnaire collecting information on their age, sex, smoking status, aerobic physical exercise and physician-prescribed asthma therapy. According to the international recommendations of the Global Initiative for Asthma (GINA), asthma severity grade was assessed according to the level of treatment needed in order to achieve the optimal disease control (Table 1) [17].

**Table 1:** Asthma severity classification according to the treatment needed to achieve optimal disease control (adapted from GINA international guidelines) [17]

STEP 1 intermittent	STEP 2 mild	STEP 3 moderate persistent	STEP 4 severe persistent	STEP 5 severe difficult to treat
low dose ICS-SABA as needed	daily low dose ICS, or daily leukotriene antagonist, or low dose ICS-SABA as needed (> 2/month)	low dose ICS-LABA, or medium dose ICS, or low dose ICS + leukotriene antagonist	medium dose ICS-LABA, or high dose ICS, add-on tiotropium, or add-on leukotriene antagonist	high dose ICS-LABA ± add-on tiotropium and/or biologic drugs and/or oral steroids

*ICS*= Inhaled corticosteroids; *SABA*= Short acting beta2 agonist; *LABA=* Long acting beta2 agonist

#### Asthma control assessment

Asthma control was assessed through ACT, a validated 5 item questionnaire which provides a snapshot on the degree of asthma control achieved over the past 4 weeks [18, 19]. Copy of the ACT questionnaire used for this study can be accessed elsewhere [18]. The overall ACT score attained by answering each of the five questions classifies asthma control as follows:

- 20–25: well controlled;
- 16–19: partially controlled;
- ≤ 16: uncontrolled

In the presence of a score < 16 the patient was recommended to refer to his GP/medical consultant as soon as possible.

#### Statistical analysis

Numbers and percentages of each variable (age, sex, smoking status, aerobic physical exercise, ACT, asthma level, habitual asthma therapy) were reported. Furthermore, the mean, standard deviation, median and range were calculated for age and ACT. A multinomial logistic regression model was fitted to investigate the risk of uncontrolled (ACT 16–19) as well as poorly controlled (ACT< 16) asthma compared to controlled asthma (Base = ACT 20+), adjusting for sex, age, asthma treatment regimen (regular vs. non-regular) and asthma level (coded from 1 to 5). Results were expressed as relative risk ratios (RRR) with 95% confidence interval (95%CI).

Asthma coded as “unclassified” was categorized as missing. All missing data were excluded, and complete case analysis was performed.

Analysis was carried out with Stata 14.2 (Stata Corporation, College Station, Texas, USA).

## Results

Overall 57 community pharmacies (41% out of all pharmacies within the catchment area of Verona health district) participated to the study, with 671 asthma patients consecutively recruited during the study period. Complete data were available for 584 out of 671 patients enrolled. Patients’ demographic information can be seen in Table 2. Study subjects were predominantly females (54%) and had a mean age of 51 ± 19 years, with 53% of them being older than 50. The mean age of female patients (52.1 years) was slightly higher than males’ (48.6 years). Most study subjects were non-smokers (54.4%), 19.2% were current smokers, and 26.4% ex-smokers. Although never-smokers were predominantly females (60.0% females vs. 48.3% males), the proportion of ex-smokers was higher among males (30.8% males vs. 22.8% females).

**Table 2:** Distribution of variables by sex of patients. Number (N); column percentage (column %); mean ± standard deviation (SD); median, Interquartile range (IQR); M = missing values; ACT= Asthma Control Test

FACTORS	STRATA	TOTAL	MALES	FEMALES
		Column %		
**N. pharmacies**		57		
**Sex** (M: 87)	**Female**	316 (54.1)		
	**Male**	268 (45.9)		
**Age** (years) (M: 86)	**Mean ± SD**	51 ± 19.9	48.6 ± 20.7	53.1 ± 19.0
	**Median** (IQR)	53 (36–68)	48.5 (33–67)	55 (39.3–68)
	**< 36**	144 (24.6)	80 (29.9)	63 (19.9)
	**36–50**	128 (21.9)	63 (23.5)	65 (20.6)
	**51–65**	144 (24.6)	54 (20.2)	90 (28.5)
	**66+**	169 (28.9)	71 (26.5)	98 (31.0)
**Smoking status** (M: 145)	**Not smoker**	286 (54.4)	113 (48.3)	171 (60.0)
	**Current smoker**	101 (19.2)	49 (20.9)	49 (17.2)
	**Ex Smoker**	139 (26.4)	72 (30.8)	65 (22.8)
**Aerobic exercise** (M: 192)	**No**	183 (38.2)	70 (35.7)	92 (38.2)
	**Yes**	292 (61.0)	125 (63.8)	146 (60.6)
	**Unclassified**	4	1 (0.5)	3 (1.2)
**ACT** (M: 6)	**Mean ± SD**	18.5 ± 5.2	18.7 ± 4.8	18.9 ± 4.8
	**Median** (IQR)	20 (15–23)	20 (16–23)	20 (15.8–23)
	**20+** (controlled)	336 (50.5)	137 (51.7)	171 (54.5)
	**16–19** (uncontrolled)	148 (22.3)	67 (25.3)	65 (20.7)
	**< 16** (poorly controlled)	181 (27.2)	61 (23.0)	78 (24.8)
**Asthma** **Level** (M: 96)	**1 (intermittent)**	76 (13.2)	31 (12.7)	44 (14.9)
	**2 (mild)**	129 (22.4)	50 (20.5)	79 (26.7)
	**3 (moderate persistent)**	219 (38.1)	109 (44.7)	108 (34.7)
	**4 (sever persistent)**	86 (15.0)	34 (13.9)	32 (10.8)
	**5 (severe difficut asthma)**	17 (3.0)	1 (0.4)	5 (1.7)
	**Unclassified**	48 (8.4)	19 (7.8)	28 (9.5)
**Regular therapy** (M: 148)	**No**	42 (8.0)	16 (7.3)	26 (9.6)
	**Yes**	481 (92.0)	204 (92.7)	246 (90.4)

Regular aerobic exercise was reported by 62% participants, with equal distribution by sex. Most participants (92%) declared to assume anti-asthmatic treatment on a regular basis, with a rather homogeneous distribution between females and males.

When considering the ACT score, asthma was controlled in 50.5% patients (51.7% males vs. 54.5% females), partially controlled in 22.3% of them (25.3% males vs. 20.7% females) and uncontrolled in the remaining 27.2% patients (23% males vs. 24.8% females).

The stratification of patients by asthma severity was as follows (Table 2):

- *Intermittent*: 76 patients (13.2%);
- *Mild persistent*: 129 patients (22.4%);
- *Moderate persistent*: 219 patients (38.1%);
- *Severe persistent*: 86 patients (15%);
- *Severe difficult asthma*: 17 patients (3%);
- *Unclassified*: 48 patients (8.4%).

The proportion of patients with mild asthma was higher among females (26.7%) than males (20.5%), whereas the prevalence of moderate (44.7% vs. 34.7%) and severe persistent (13.9% vs. 10.8%) disease was higher among males.

**Fig. 1:**
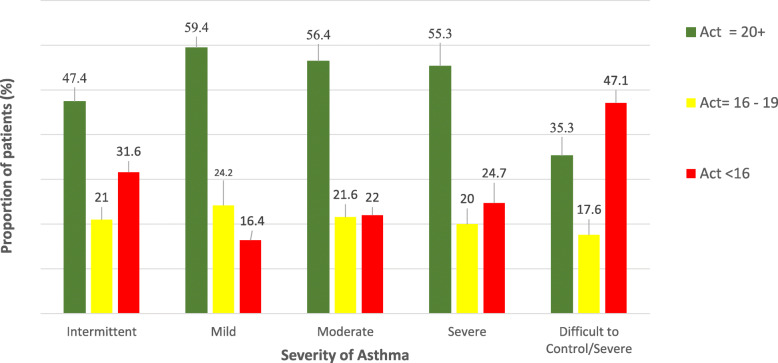
Distribution of patients by asthma control test (ACT) and severity of asthma

Table 3 and Fig. 1 report the level of asthma control by severity of the disease. The mean ACT score was rather consistent across the various categories of disease severity, being lower only for severe asthma (level 5). By contrast, whilst the pattern of mean ACT was homogeneous among females, it increased by disease severity in males (Table 3).

**Table 3:** Distribution of asthma control test (ACT by severity of asthma and sex od patients. Number (N); row percentages (row %); Mean ± standard deviation (SD)

FACTORS	STRATA	ACT			
		Mean ± SD	20+	16–19	< 16
			Row %		
**All PATIENTS**					
**Age**	< 50 years	19.2 ± 4.8	7 (2.7)	114 (43.9)	139 (53.5)
	50+ years	18.7 ± 4.6	16 (5.0)	135 (2.2)	169 (52.8)
**Smoking status**	Non-smoker	18.9 ± 4.63	9 (3.2)	123(43.8)	149 (53.0)
	Smoker	19.6 ± 4.6	3 (3.0)	37 (37.0)	60 (60.)
	Ex-smoker	19.6 ± 4.4	3 (2.2)	56 (40.3)	80 (57.6)
**Asthma Level**	**1**	19.0 ± 5.1	36 (47.4)	16 (21.1)	24 (31.6)
	**2**	19.6 ± 4.3	76 (59.4)	31 (24.2)	21 (16.4)
	**3**	19.3 ± 4.5	123 (56.4)	47 (21.6)	48 (22.0)
	**4**	19.3 ± 4.7	47 (55.3)	17 (20.0)	21 (24.7)
	**5**	16.2 ± 5.8	6 (35.3)	3 (17.7)	8 (47.1)
	**Unclassified**	18.8 ± 4.3	21 (46.7)	14 (31.1)	10 (22.2)
**MALES**					
**Asthma Level**	**1**	1.7 ± 5.2	13 (41.9)	9 (29.0)	9 (29.0)
	**2**	19.0 ± 4.5	26 (52.0)	16 (32.0)	8 (16.0)
	**3**	19.2 ± 4.7	64 (58.7)	18 (16.5)	27 (24.8)
	**4**	20.5 ± 4.1	21 (63.6)	7 (21.2)	5 (15.2)
	**5**	8	0	0	1
	**Unclassified**	18.9 ± 3.9	8 (47.1)	6 (35.3)	3 (17.7)
**FEMALES**					
**Asthma Level**	**1**	18.1 ± 5.1	23 (52.3)	6 (13.6)	15 (34.1)
	**2**	19.9 ± 4.2	50 (64.1)	15 (19.2)	13 (16.7)
	**3**	19.5 ± 4.3	59 (55.1)	27 (25.3)	21 (19.6)
	**4**	19.2 ± 5.3	19 (59.4)	4 (12.5)	9 (28.2)
	**5**	17.2 ± 3.9	2 (40.0)	2 (40.0)	1 (20.0)
	**Unclassified**	18.7 ± 4.7	13 (46.4)	8 (28.6)	7 (25.0)

A lower proportion of patients with poorly controlled asthma (ACT< 16) was found among those affected by mild disease (16.4%), with similar pattern of distribution between females and males. Among patients with uncontrolled asthma (ACT =16–19), the proportion with moderate persistent disease was higher in females (25.3%) than males (16.5%), whereas the percentage of patients with severe persistent disease was higher among males (21.2%) than females (12.5%). Among patients with poorly controlled asthma (ACT< 16) the percentage with moderate persistent disease was higher among males (24.8%) than females (19.6%), whereas more females had severe persistent asthma (29.2% females vs. 15.2% males).

Table 4 shows the results of the multinomial logistic regression analysis investigating the risk of uncontrolled (ACT 16–19) and poorly controlled (ACT < 16) vs. controlled asthma (base), adjusting for the effect of sex, age, treatment adherence and asthma severity level. As can be seen, patients under regular asthma treatment course (RRR = 0.33; 95%CI: 0.15; 0.77) were less likely to have an ACT< 16 compared to those not taking medications regularly.

**Table 4:** Multinomial logistic regression analysis for the probability of asthma control test (ACT) of 16–19 and < 16 over ACT > 19 (base). Multiple regression model adjusted for sex, age, reported treatment adherence and asthma severity. Number (N); row percentage (%); relative risk ratio (RRR) with 95% confidence intervals (95%CI). 444 complete (case analysis) observations

FACTORS	STRATA	ACT 20+ (Base)	ACT 16–19		ACT < 16	
		N (%)	N (%)	RRR (95%CI)	N (%)	RRR (95%CI)
**Regular therapy**	**No**	17 (40.5)	11 (26.2)	Reference	14 (33.3)	reference
	**Yes**	103 (21.6)	98 (20.6)	0.45 (0.20; 1.22)	276 (57.9)	0.33 (0.15; 0.77)
**Asthma level**	**1**	36 (47.4)	16 (21.1)	reference	24 (31.6)	reference
	**2**	76 (59.4)	31 (24.2)	1.44 (0.56; 3.72)	21 (16.4)	0.46 (0.21; 1.00)
	**3**	123 (55.3)	47 (21.6)	1.48 (0.60; 3.68)	48 (22.0)	0.58 (0.29; 1.16)
	**4**	47 (55.3)	17 (20.0)	1.10 (0.38; 3.24)	21 (24.7)	0.58 (0.25; 1.38)
	**5**	6 (35.3)	3 (17.7)	4.16 (0.49; 35.65)	8 (47.1)	1.63 (0.21; 13.0)

## Discussion

Our study explored the assessment of asthma control in a community pharmacy setting, using the ACT instrument, a standardized and validated five-item questionnaire measuring asthma control [20]. Although being easy-to-use and accurate in providing immediate disease control evaluation, ACT is still underused by general practitioners (GPs) and medical consultants [21].

In the present study the local pharmacies detected an unsatisfactory asthma control in the general population of the Verona Health District. In particular, based upon ACT score, 50.5% patients had controlled disease, 22.3% poorly controlled and 27.2% uncontrolled. A variable proportion of patients with uncontrolled asthma was observed at every level of severity of the disease, although more frequently among those affected by mild persistent asthma. Most patients (92%) reported regular compliance with prescribed treatment. Nonetheless, the main determinant of poor asthma control (ACT< 16) was treatment adherence, after removing the effect of all other factors (including disease severity).

Although this result may be intuitive, asthma control still represents a challenge worldwide [7, 8] and the community pharmacies have been identified as a potential relevant partner in sharing this challenge with the other health care providers, monitoring treatment compliance for asthma, a critical aspect to control the disease [14]. Pharmacists can in fact offer patients a first point of contact with the health care services, easy to access for disease counselling. In his way, community pharmacies somehow compensate the obstacles of patients to access hospital care as well as the limited time for consultations dedicated in GPs settings [14, 16]. The local pharmacies could also play a unique role in the assessment of asthma control by combining the ACT and asthma medicine records as a proxy of treatment compliance.

Although the involvement of primary care (particularly pharmacists) in asthma control is recommended by current international guidelines [19], only a few studies have been conducted on this topic and none has been carried out in Italy [22–25].

According to our results the level of asthma control assessed by ACT was overall higher in comparison with studies using the same tool but conducted in a medical setting in Italy [5–8] or in community pharmacies of other European countries [22–25]. Some reasons may account for this discrepancy. The mean age of our study population was > 50 years, whilst in previous studies reporting a worse asthma control, a higher proportion of younger patients was recruited. It has been previously found that the prevalence of uncontrolled asthma is higher among young adults and adolescents [26].

Adult or elderly patients tend to be more familiar and comfortable with the local pharmacies, hence they receive more frequently advice on the need of regular asthma therapy.

However, the older age of our patients raises plausible concerns of the differential diagnosis with other chronic respiratory conditions, particularly chronic obstructive pulmonary disease (COPD); in which case the ACT may provide an unreliable score as it is not a validated instrument for obstructive respiratory syndromes other than asthma.

Although low treatment adherence was the only determinants of poorly controlled asthma, patients recruited in the present study showed a surprisingly high overall crude rate of treatment adherence (92%). This finding is quite unexpected, when considering the actual data from the Italian Medicine Agency (AIFA), which report an overall poor treatment adherence for asthma [27]. Furthermore, the adherence rate, when objectively evaluated, seems to be independent of the disease severity [28, 29], so that the prevalence of patients with moderate and severe persistent asthma in our population cannot be truly considered a potential explanation for our observation. More likely it reflects the typical overestimation of patients when interviewed about their compliance with therapy, already reported in the open literature [30, 31]. Although asthmatic patients can be accurately identified by the asthma exemption code, patient self-reports may bias the evaluation of their treatment adherence, which should be ideally assessed by medicine records of community pharmacies, an information not available for the present study.

The lack of asthma control was more frequent among patients with mild persistent asthma in the present study. This finding is not surprising, as in our previous pilot study we reported a 31% prevalence of uncontrolled asthma in a GP setting [8]. It is plausible that the presence of intermittent symptoms led these patients to a treatment on demand, with a consequent overuse of beta-2 short agonists and underuse of inhaled steroids. Moreover, these patients usually prefer self-medication than regular follow-up by their GPs or by specialist medical consultants. However, the risk of fatal asthma is still possible with mild persistent disease, as recently reported [32].

Whilst the positive results of this study suggest the feasibility of asthma control assessment at the local pharmacy level, an overall inclusion of the community pharmacist is a challenging target, as not all of them may be keen in being involved in a similar health plan, rating it demanding and time consuming, particularly in periods of the year of high morbidity and intense access to pharmacies [33]. Therefore, in addition to careful selection of well trained, motivated community pharmacists, within a structured health plan, value-based incentives (VBI) programs may also be considered. Similar to other health care settings (e.g. GP practices), financial incentives could be granted to pharmacies to accomplish quality health outcomes in patients [15]. In addition of being an accessible setting for asthma control assessment, the local pharmacies could also provide counselling on disease control outside health care settings. Trained pharmacists would have also the opportunity to teach patients about the disease and the proper use of medical devices, thus facilitating enrolled patients’ [34]. Moreover, the local pharmacy could also be an optimal setting to deliver spirometry tests for a fee. However, despite charging patients for spirometry could further motivate the participation of pharmacists in asthma control plans, similar measures are still open to debate, since the interpretation of spirometry entails specific competences that should be limited to trained and certified pharmacies [34]. Finally, community pharmacies could also provide counselling on smoking cessation, as in our study population one out of five asthmatic patients was a smoker.

## Conclusions

This study found an unsatisfactory asthma control in the general population of the Verona Health District. Patients with treatment compliance were less likely to have a poorly controlled asthma, adjusting for other actors (age, sex and disease severity). Patients with chronic conditions may face barriers to access burdened health care services in high income countries. The local pharmacy is an underused yet widely accessible primary care setting potentially useful to promote health in the general population. Due to the high prevalence of asthma in the general population, the inclusion of allied health professional as local pharmacists may represent a step forward to be considered with the view of improving the control of the disease. Local pharmacies may provide critical cost-effective support to screen patients for their risk of asthma exacerbations, increase their knowledge of the disease, assess asthma control, improve inhalation techniques and follow up their treatment adherence, which should rely on pharmacies’ medicine records rather than on patients’ self-reports. However, besides carefully selecting well trained and motivated community pharmacists, VBI programs may also be considered to incentivize pharmacist to accomplish quality health outcomes in their patients served.

## Data Availability

The datasets generated and analysed during the current study are not publicly available, since they were purposively collected by the authors for the present study, but are available from the corresponding author on reasonable request.

## Abbreviations

ACT: Asthma control test
CI: Confidence interval
COPD: Chronic obstructive pulmonary disease
GINA: Global initiative for asthma
GP: General practitioner
NHS: National health service
RRR: Relative risk ratio
VBI: Value-based incentive
